# Restricted N-glycan Conformational Space in the PDB and Its Implication in Glycan Structure Modeling

**DOI:** 10.1371/journal.pcbi.1002946

**Published:** 2013-03-14

**Authors:** Sunhwan Jo, Hui Sun Lee, Jeffrey Skolnick, Wonpil Im

**Affiliations:** 1 Department of Molecular Biosciences and Center for Bioinformatics, The University of Kansas, Lawrence, Kansas, United States of America; 2 Center for the Study of Systems Biology, School of Biology, Georgia Institute of Technology, Atlanta, Georgia, United States of America; Fox Chase Cancer Center, United States of America

## Abstract

Understanding glycan structure and dynamics is central to understanding protein-carbohydrate recognition and its role in protein-protein interactions. Given the difficulties in obtaining the glycan's crystal structure in glycoconjugates due to its flexibility and heterogeneity, computational modeling could play an important role in providing glycosylated protein structure models. To address if glycan structures available in the PDB can be used as templates or fragments for glycan modeling, we present a survey of the N-glycan structures of 35 different sequences in the PDB. Our statistical analysis shows that the N-glycan structures found on homologous glycoproteins are significantly conserved compared to the random background, suggesting that N-glycan chains can be confidently modeled with template glycan structures whose parent glycoproteins share sequence similarity. On the other hand, N-glycan structures found on non-homologous glycoproteins do not show significant global structural similarity. Nonetheless, the internal substructures of these N-glycans, particularly, the substructures that are closer to the protein, show significantly similar structures, suggesting that such substructures can be used as fragments in glycan modeling. Increased interactions with protein might be responsible for the restricted conformational space of N-glycan chains. Our results suggest that structure prediction/modeling of N-glycans of glycoconjugates using structure database could be effective and different modeling approaches would be needed depending on the availability of template structures.

## Author Summary

An N-glycan is a carbohydrate chain covalently linked to the side chain of asparagine. Due to the flexibility of carbohydrate chains, it is believed that the N-glycan chains would not have a well-defined structure. However, our survey of N-glycan structures in the PDB shows that the N-glycan structures found on the surfaces of homologous glycoproteins are significantly conserved. This suggests that the interaction between the carbohydrate and the protein structure around the glycan chain plays an important role in determining the N-glycan structure. While the global N-glycan structures found on the surfaces of non-homologous glycoproteins are not conserved, the conformations of the carbohydrate residues that are closer to the protein appear to be more conserved. Our analysis highlights the applicability of template-based approaches used in protein structure prediction to structure prediction and modeling of N-glycans of glycoproteins.

## Introduction

Glycosylation represents one of the most important post-translational modifications [1], [2] and is ubiquitous in all domains of life. The glycosylation machinery is largely conserved in eukaryotes, and more than 50% of all eukaryotic proteins are expected to be glycosylated [3], [4]. An oligosaccharide moiety in a glycoprotein, referred to as a glycan, comes in a diversity of sequences and structures and plays critical roles in a vast array of biological processes [1]. The N-glycosylation pathway is the most common pathway in which an oligosaccharide is covalently attached to the side chain of asparagine [2]. In general, such an oligosaccharide appendage masks the protein surface, protecting the glycoprotein from degradation and nonspecific protein-protein interactions (reviewed in [5]–[7]). N-glycosylation also alters the biophysical properties in the vicinity of the glycosylation site and affects the folding rates and the thermal stability of the protein [8], [9]. Some N-linked oligosaccharides (N-glycans) are directly involved in specific molecular recognition events; e.g., lectins and antibodies can recognize specific N-glycans on viral envelope glycoproteins such as HIV gp120 [10]–[13].

The impact of glycosylation on the structure of the parent protein and *vice versa* has been of great interest in structural glycobiology [8], [14]–[17]. At this time, however, an understanding of which glycans are important components in protein function and how to modify these glycans to optimize the protein properties of interest remain an enigma. Therefore, knowledge of the structure and dynamics of N-glycans is central to understanding protein-carbohydrate recognition and its role in protein-protein interactions. An oligosaccharide chain is flexible in solution and has an ensemble of diverse conformations rather than a single well-defined structure [18]–[20]. The inherent flexibility of oligosaccharides often hinders crystallographic structure determination, and there are only a few crystal structures of oligosaccharides longer than 2–3 residues in the Cambridge Structure Database [21]. In contrast, there are many more crystal structures of glycoconjugates in the Protein Data Bank (PDB) [22], suggesting that the presence of the protein may reduce the conformational freedom of oligosaccharides or even favor a certain conformation over others [23]. For example, the N-glycan conformations in the crystal structures of the Fc domain [24]–[30] exhibit remarkable similarity (Figure S1 in Supporting Information), suggesting that the protein's structure around the glycan has an influence on the glycan's conformation.

The number of PDB entries containing carbohydrates has been steadily increasing, but obtaining the complete N-glycan structure remains challenging [23]. Mass spectrometric mapping of N-glycosylation sites is becoming common [4], providing information about glycosylation sites as well as the relative abundance of different glycoforms. In this context, computational modeling of N-glycan structures is an appealing approach to provide glycosylated protein structure models. In particular, a computational approach that can combine known glycoprotein structures and glycosylation information (i.e., glycosylation site, primary glycan sequence, and linkage information) would be very useful in a variety of applications in glycoscience. For successful template-based glycan structure modeling, it is essential to understand the conformational variability of an oligosaccharide chain when it is glycosylated. In addition, the influence of the protein residues around the glycosylation site can provide valuable insight into the design of new computational approaches that are optimized for glycoconjugates. Several structural database surveys have investigated the general features of N-glycosylation in terms of oligosaccharide and protein structures [14], [23], [31]–[35]. In these earlier studies, however, the oligosaccharide conformations were analyzed in terms of individual glycosidic torsion angles, making it difficult to recognize the actual structural variability of glycans *en bloc*. To the best of our knowledge, the conformational variability of N-glycans using the three-dimensional (3D) structures in the PDB has not been studied.

In this work, using the PDB crystal structures that contain N-glycans, we examined the conformational variability in various N-glycans. Using *Glycan Reader* [36], an automatic sugar recognition algorithm that we developed, all N-linked glycoprotein structures were obtained from the PDB and sorted by their N-glycan sequence. PDB entries with more than 3 Å resolution were excluded and N-glycan sequences with less than 20 PDB entries were also excluded, resulting in 35 N-glycan sequences (see the full list in Table S1 in Supporting Information). Using random background conformations of each N-glycan sequence, the statistical significance of glycan structural similarity was estimated. The N-glycan structures in the PDB show statistically significant similarity when the local structure around the protein is conserved. When the local protein structures are different, overall N-glycan structures are not conserved, but their internal substructures appear to be strongly conserved due to the proximity to the protein. Our results highlight the applicability of template-based approaches used in protein structure prediction to the structure prediction and modeling of N-glycans of glycoproteins. Although the N-glycan sequences examined in this work mostly represent oligomannose-type glycans due to the limited numbers of crystal structures of complex- and hybrid-type glycans, the conclusions might be applicable to other glycoconjugates' glycan sequences.

## Results

**Figure 1:**
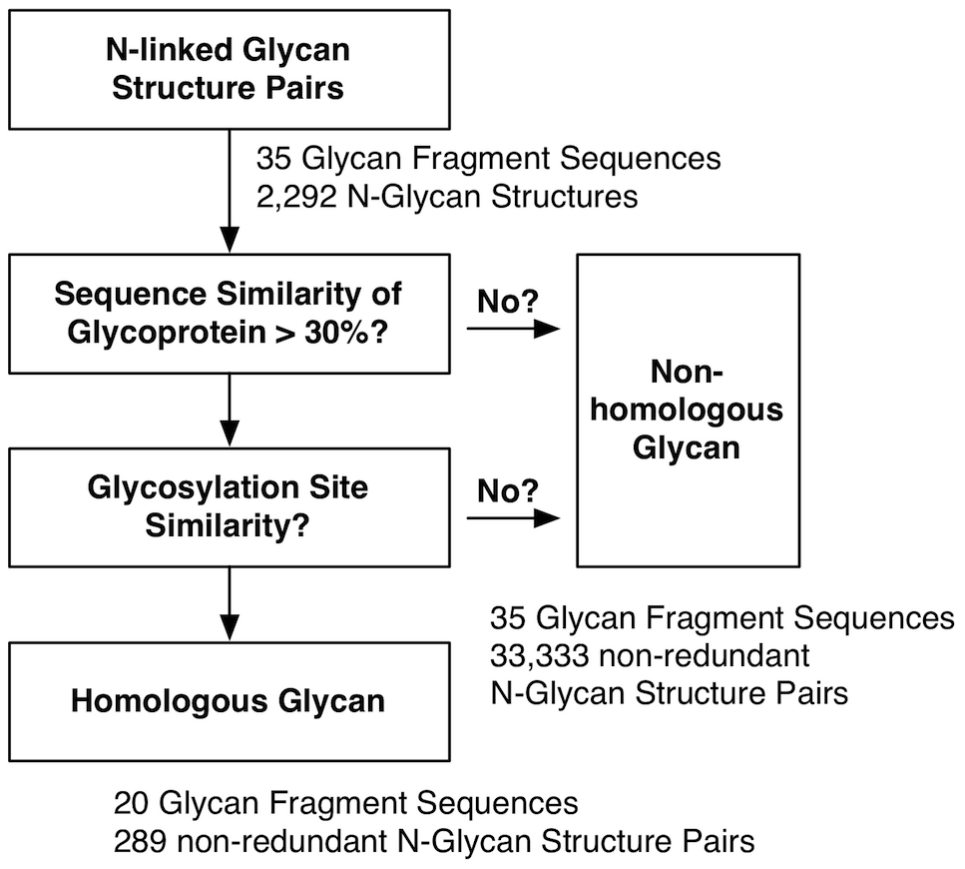
Protocol for building the N-glycan structure dataset.

Because glycan sequences have branches and different linkages between monomers, alignment of glycan structures with different sequences is challenging and, to the best of our knowledge, there is no such an alignment tool for glycans. Therefore, in this study, pairwise structure similarity is measured using the root-mean-squared deviation (RMSD) among glycan structures having the *identical* glycan sequence. Assuming that homologous protein structures share similar surface features, the structural similarity of glycans found on homologous proteins would provide insight into the influence of the protein structure on the N-glycan structure. Therefore, N-glycan structure pairs with the identical glycan sequence are designated as “homologous” or “non-homologous” depending on the sequence similarity of their parent proteins (with a sequence similarity of 30% as a cutoff). Unless stated explicitly, highly homologous pairs (sequence similarity ≥90%) as well as redundant structure pairs were excluded from the analysis. There are a total of 289 homologous and 33,333 non-homologous glycan structure pairs in the final dataset (see Figure 1 and Methods for details). In this section, N-glycan structural similarity is examined and its statistical significance is estimated using random background conformations of each N-glycan sequence (see Methods for details). The structural similarity of the N-glycans is then discussed in terms of the protein's structure as well as the structural rigidity of the oligosaccharide regions that are closer to the glycosylation site on the protein.

### N-glycan structures on the surface of homologous proteins are significantly conserved

**Figure 2:**
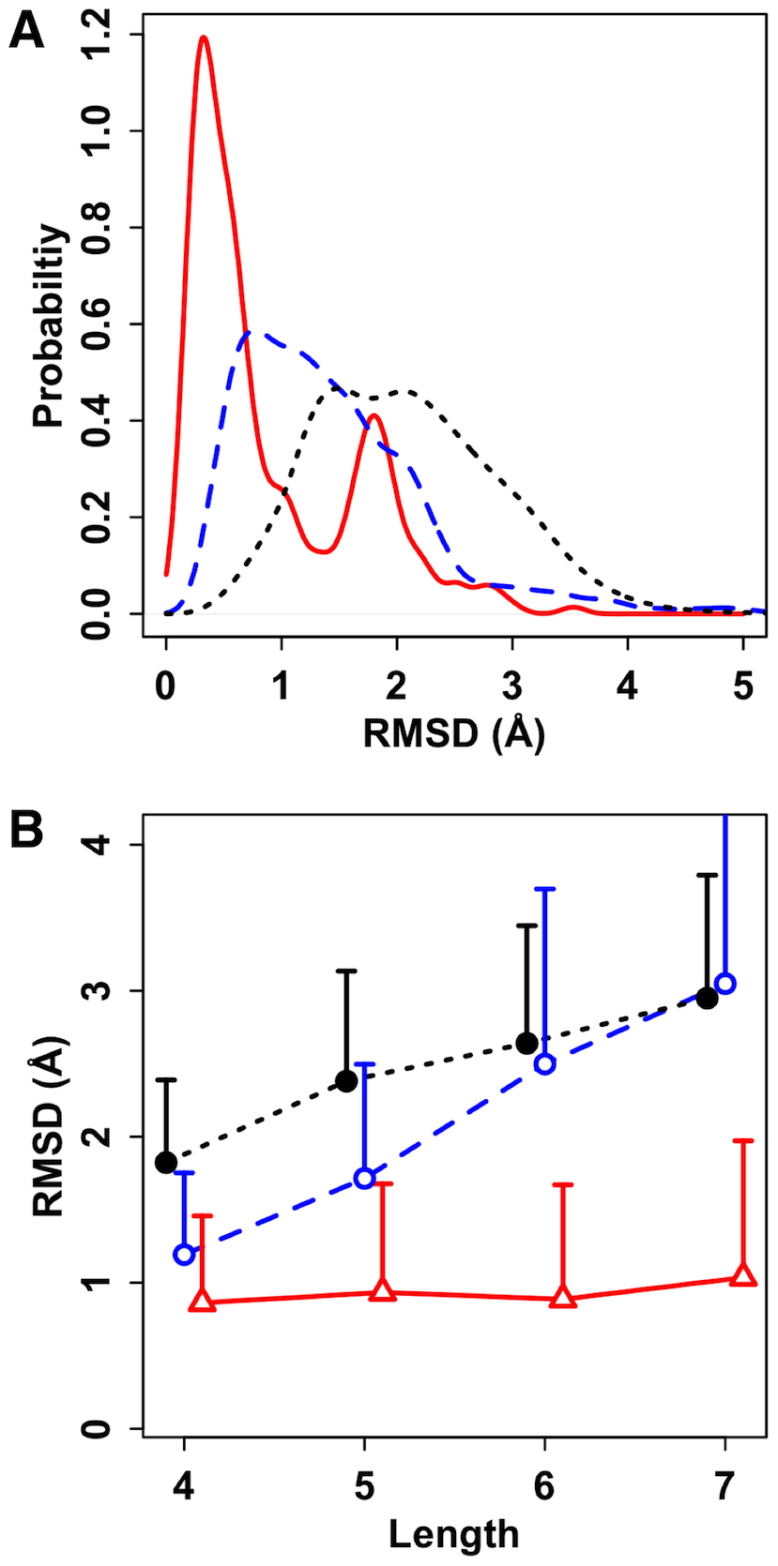
N-glycan structure similarity. (A) The RMSD distributions from the homologous (red), non-homologous (blue), and random glycan structure pairs (black). (B) Length dependence of average RMSD values from homologous (red), non-homologous (blue), and random glycan structure pairs (black). The length of a glycan chain is defined as the number of residues in the glycan chain. Error bars are the standard deviations and only the upper sides are displayed for clarity. Each data point is slightly shifted for clarity. Red and blue colors represent the homologous and non-homologous N-glycans, and the same color scheme is adopted throughout the figures unless stated otherwise.

The structural similarities of the N-glycans are measured by calculating the glycan RMSD after alignment of the oligosaccharide structures using the carbohydrate ring heavy atoms. N-glycan structural similarity including their orientations with respect to the protein is discussed separately below. Figure 2 shows the RMSD distributions of the N-glycan structure pairs in the PDB and random conformation pool. Note that the RMSD is only measured between glycan structures having an identical sequence. The average RMSD of all PDB structural pairs are 1.4±0.8 Å. The homologous and the non-homologous N-glycan structure pairs have RMSD values of 0.9±0.8 Å and 1.4±0.8 Å, respectively. Both the homologous and non-homologous N-glycans showed smaller RMSD values compared to those in the random glycan structure pool whose RMSD is 2.4±0.8 Å (Figure 2A).

Measuring the structural similarity using RMSD is straightforward, but it is not an objective measure when comparing structures of different lengths and sequences due to its length dependence. When the average RMSD values of the N-glycans are plotted against N-glycan length, i.e., the number of carbohydrate monomers (Figure 2B), a length dependence is observed for the random background and non-homologous glycan pairs, but homologous glycan pairs do not show such a length dependence. The smaller RMSD values of the homologous N-glycan structure pairs compared to the RMSD values of the non-homologous pairs indicate that the homologous N-glycan structures are more conserved than the non-homologous N-glycan structures.

Because our dataset contains different lengths of N-glycan sequences with different branching patterns (Table S1), we converted the RMSD values to their statistical significance (*p*-values) using the random background glycan structures (see Methods for details). By deriving the statistical significance using the random background having the identical N-glycan sequence, the length dependence is effectively removed. The generalized extreme value distribution (Eq. 1 in Methods) was used to estimate the statistical significance [37], and 35 sets of parameters were determined by fitting the generalized extreme value distribution to the original RMSD distribution of the random conformational pool of each glycan sequence (see the determined parameters in Table S2 and the fitting results in Figure S2). The calculated *p*-values (Eq. 2 in Methods) represent the probability of having randomly chosen two N-glycan structures whose RMSD is smaller than the random background. A list of *p*-values and the corresponding RMSD values averaged over different sequences are given in Table 1.

**Table 1:** Statistical significance of the RMSD values for the PDB N-glycan pairs.

N-glycan length¶	p-value	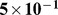	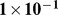	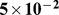	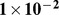
Overall	RMSD	2.4 Å	1.8 Å	1.5 Å	0.9 Å
7	RMSD	2.9 Å	1.9 Å	1.6 Å	1.2 Å
6	RMSD	2.6 Å	1.7 Å	1.5 Å	1.1 Å
5	RMSD	2.3 Å	1.4 Å	1.2 Å	0.8 Å
4	RMSD	1.8 Å	1.4 Å	1.0 Å	0.7 Å

¶ The N-glycan length is defined as the number of residues in a glycan chain.

**Figure 3:**
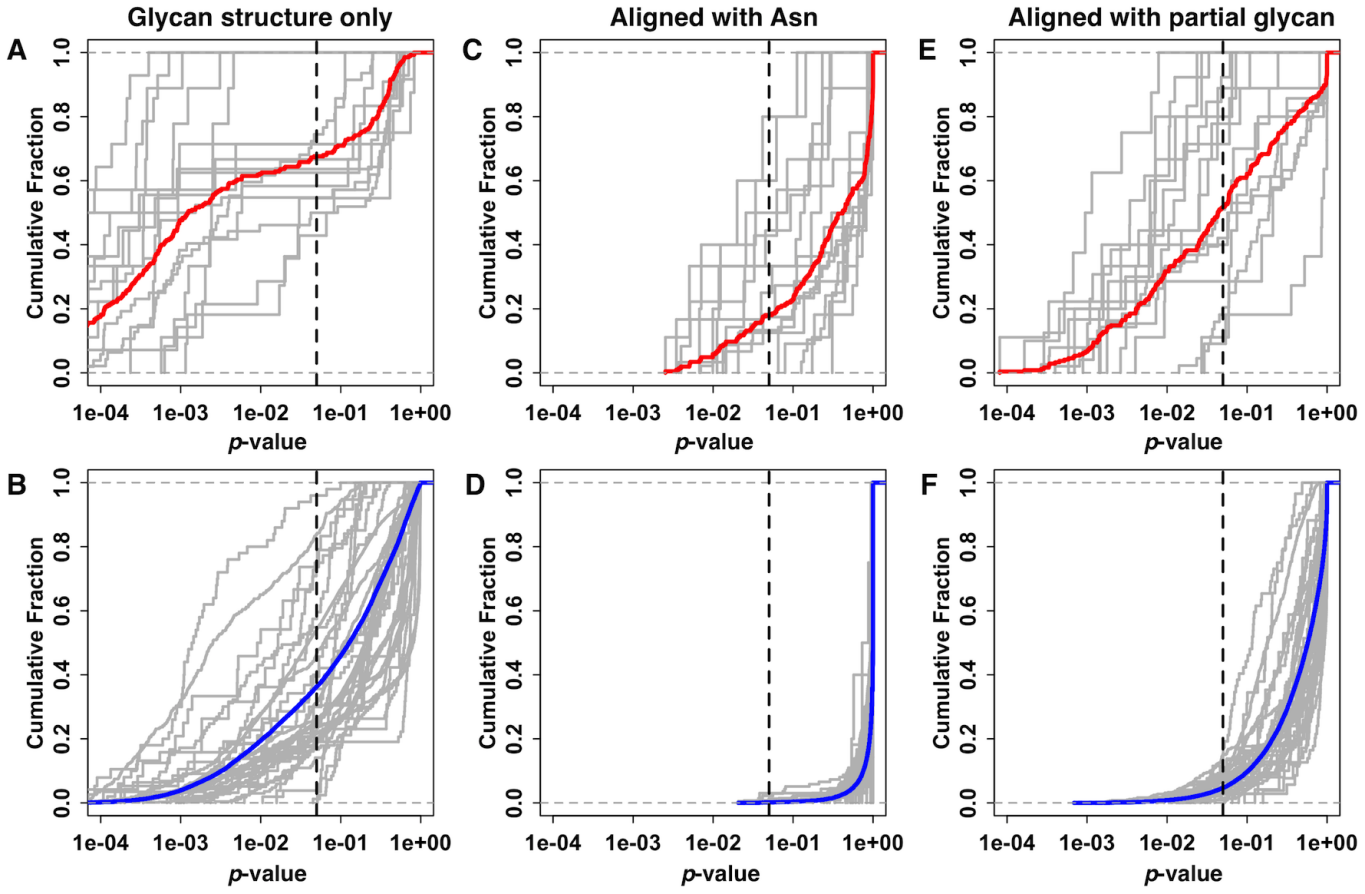
Cumulative fraction of glycan structure similarity using *p*-values. (A–B) Structural similarities of (A) homologous and (B) non-homologous glycans after alignment of glycan structures themselves. (C–D) Structural similarities of (C) homologous and (D) non-homologous glycans after alignment of glycosylated protein Asn residues. (E–F) Structural similarities of (E) homologous and (F) non-homologous glycans after alignment of the first two residues of the glycan chain. The gray lines in each plot represent the structural similarity of individual glycan sequences and the thick solid lines represent the average of cumulative fractions of all 35 N-glycan sequences. The vertical dotted line is drawn at a *p*-value of 0.05.

Figures 3A and 3B show the cumulative fraction of homologous and non-homologous glycans structure pairs as a function of their *p*-value. It is clear that about 67% of the homologous N-glycan structure pairs have a statistically significant level (*p*<0.05) of structural similarity, whereas about 36% of non-homologous N-glycan structure pairs have a statistically significant level of structural similarity. A correlation is also found between the sequence similarity of the glycoprotein and the structural similarity of the N-glycan (Figure S3). Specifically, about 81% and 91% of N-glycan structure pairs have statistically significant structure similarity when the parent proteins have sequence similarity greater than 50% and 60%, respectively. A similar analysis has been carried out independently using the global distance test (GDT) score [38] instead of RMSD, and the conclusion remains the same (Figure S4). Assuming that the proteins with similar sequences have similar surface features around the glycosylation site, such a high level of N-glycan structure similarity strongly indicates that the protein structure around the N-linked oligosaccharide plays an important role in determining the N-glycan structures.

**Figure 4:**
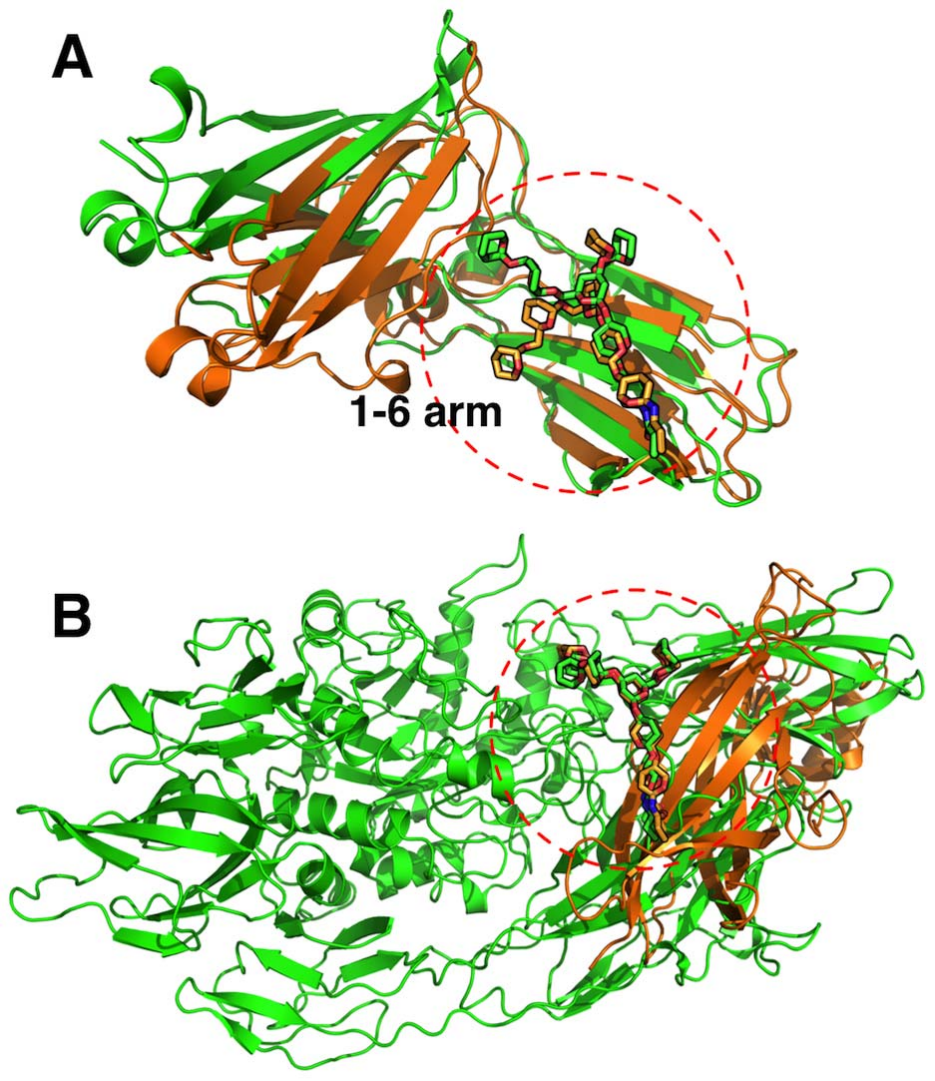
Examples of N-glycan structure pairs. (A) An example of homologous glycoproteins having dissimilar glycan structures. The IgG-Fc domain (PDB:2WAH) is drawn in green and the IgE-Fc domain (PDB:3H9Y) is drawn in orange. The RMSD of the two oligosaccharides is 2.9 Å. Hydroxyl groups of the oligosaccharides are removed for clarity. (B) An example of non-homologous glycoproteins having similar glycan structures. The beta-galactosidase (PDB:3OG2) is drawn in green and the extracellular domain of the nicotinic acetylcholine receptor 1 subunit (PDB:2QC1) is drawn in orange. The RMSD of the two oligosaccharides is 0.9 Å.

Apparently, not all homologous glycans have significant structural similarity. Figure 4A shows an example of two homologous proteins, the Fc domain of IgG (PDB:2WAH) in green and the Fc domain of IgE (PDB:3H9Y) in orange, which share a sequence similarity of about 50% and have significantly different glycan structures (RMSD of 2.9 Å and *p*-value of 0.6). The structures of these two homologous proteins around the glycosylation site are similar and well aligned. Notably, the structural difference of the N-glycans arises mainly from the terminal residues at the 1–6 branches (or 1–6 arm). The PDB:2WAH IgG-Fc domain is glycosylated with a different glycoform than typical IgG-Fc glycans whose 1–6 arm carbohydrates are tightly packed with the proteins [24]–[30]. This may explain such a different glycan conformation in PDB:2WAH.

There are some non-homologous N-glycan structure pairs that have a statistically significant level of structural similarity. Visual inspection of several examples of non-homologous glycoproteins having similar N-glycan conformations shows no apparent similar protein surface features around the N-glycans. Figure 4B shows an example of two non-homologous glycoproteins, beta-galactosidase (PDB:3OG2) in green and the extracellular domain of the nicotinic acetylcholine receptor 1 subunit (PDB:2QC1) in orange, having a significant level of structural similarity of the N-glycan (RMSD of 0.9 Å and *p*-value of 0.009). Nonetheless, the structure alignment of these two N-glycans results in a poor alignment of the parent proteins.

### N-glycan orientations with respect to the protein are diverse even in homologous glycoproteins

The relative orientation of an oligosaccharide chain with respect to the parent protein can be affected by the Asn side chain conformation and the protein conformation in the vicinity of the glycosylation site. To examine N-glycan structural variability with respect to the parent protein, the heavy atoms of the glycosylated Asn residue were used for alignment of each pair, and then the Euclidean distance of the glycan portion was measured without further alignment. Figures 3C and 3D show the cumulative fraction of structure similarity of the homologous and non-homologous glycans aligned with glycosylated Asn residues. Clearly, structural similarity is greatly reduced when the Asn residues are used for the alignment. Given the fact that glycosylation has a bias towards turns and extended regions [32], it is not surprising that even homologous N-glycans show reduced structural similarity when the Asn residues are used for the alignment.

The observations so far indicate that a comparative modeling approach for N-glycan structures would successfully predict the N-glycan structure itself, especially when the homologous N-glycan templates are present in the PDB, but finding the global orientation of the glycan with respect to the protein would remain challenging. Such difficulties can be significantly alleviated when a partial glycan structure is available. In fact, there are large numbers of partial N-glycan structures available in the PDB, probably due to the removal of glycans prior to structural studies, due to crystallization conditions, or due to missing electron density resulting from flexible glycan structures. For example, as of December 2011, there were 2,517 PDB entries and 10,769 N-linked glycan chains in the RCSB database; 84% (9,027 chains) had partial glycan structures with less than two carbohydrate units and 15% (1,394 chains) of such partial structures showed their parent protein sequence similarity less than 50%. Assuming that one can find such partial glycan structures, Figures 3E and 3F show the cumulative structural similarity of the N-glycans when the first two carbohydrate units in the glycan chains are aligned. Both the structural similarities of the homologous and non-homologous N-glycan structures (especially the former) significantly increased, suggesting that the conformations of glycosylated Asn residues and the first few carbohydrates of the N-glycan are important in determining the N-glycan orientations.

### The local structure around the glycoprotein influences the N-glycan conformation

What makes homologous N-glycan structures conserved compared to non-homologous N-glycans or random background? Possibly, the protein structures around the glycan may provide a steric barrier, thus restricting the conformational freedom of N-glycans nearby. In addition, specific protein-carbohydrate interactions may play an important role in favoring a certain conformation of the oligosaccharides. If local protein structure around the N-glycan is directly correlated with the N-glycan structure similarity, such information provides valuable criteria in N-glycan structure modeling.

**Figure 5:**
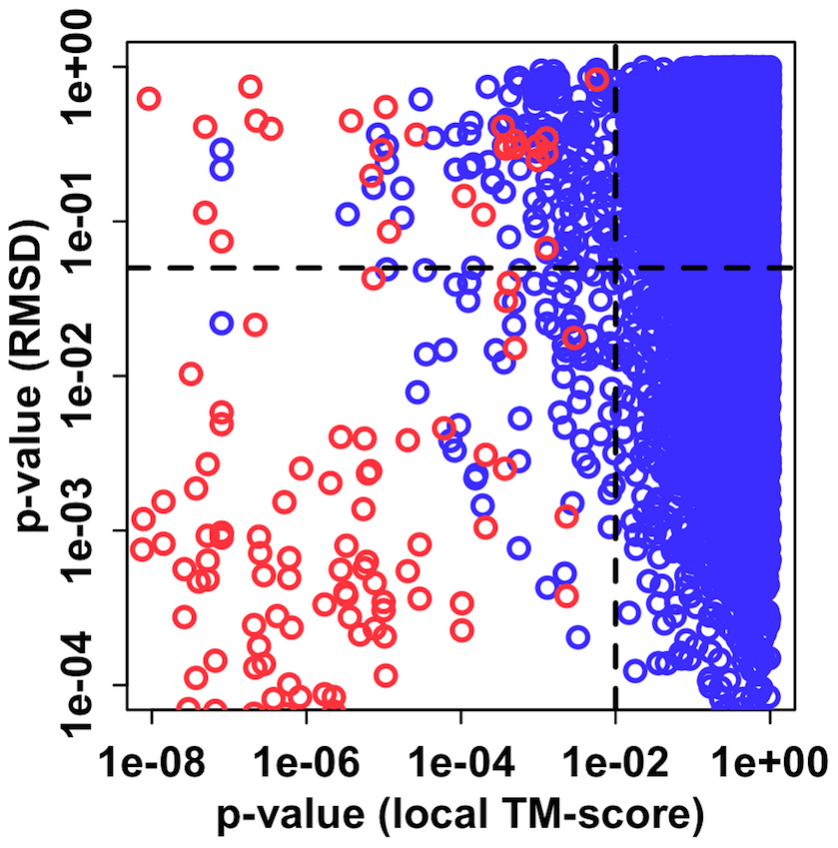
Correlation of local protein structure around the N-glycan and the N-glycan conformation. Red circles represent homologous glycan structure pairs and the blue circles are for non-homologous glycan structure pairs.

Figure 5 shows the correlation between the local protein structure around the glycan chain and the N-glycan structure similarity. As expected, most homologous glycoproteins have similar local protein structures around the glycan chain. However, some homologous N-glycan structure pairs adopt significantly different conformations while their local protein structures are similar (*p*-RMSD>0.05 and *p*-local<0.01). Visual inspection of such structures shows that the structural differences are mainly due to the terminal residues, especially ones in the 1–6 branches, similar to the case in Figure 4A. The increased flexibility of the 1–6 linkage is not surprising because the 1–6 glycosidic linkage contains three rotatable torsional angles (compared to two for other glycosidic linkages), and the flexibility of the 1–6 linkage has been well documented by other experimental, computational, and structural database surveys [34], [39]–[42].

**Figure 6:**
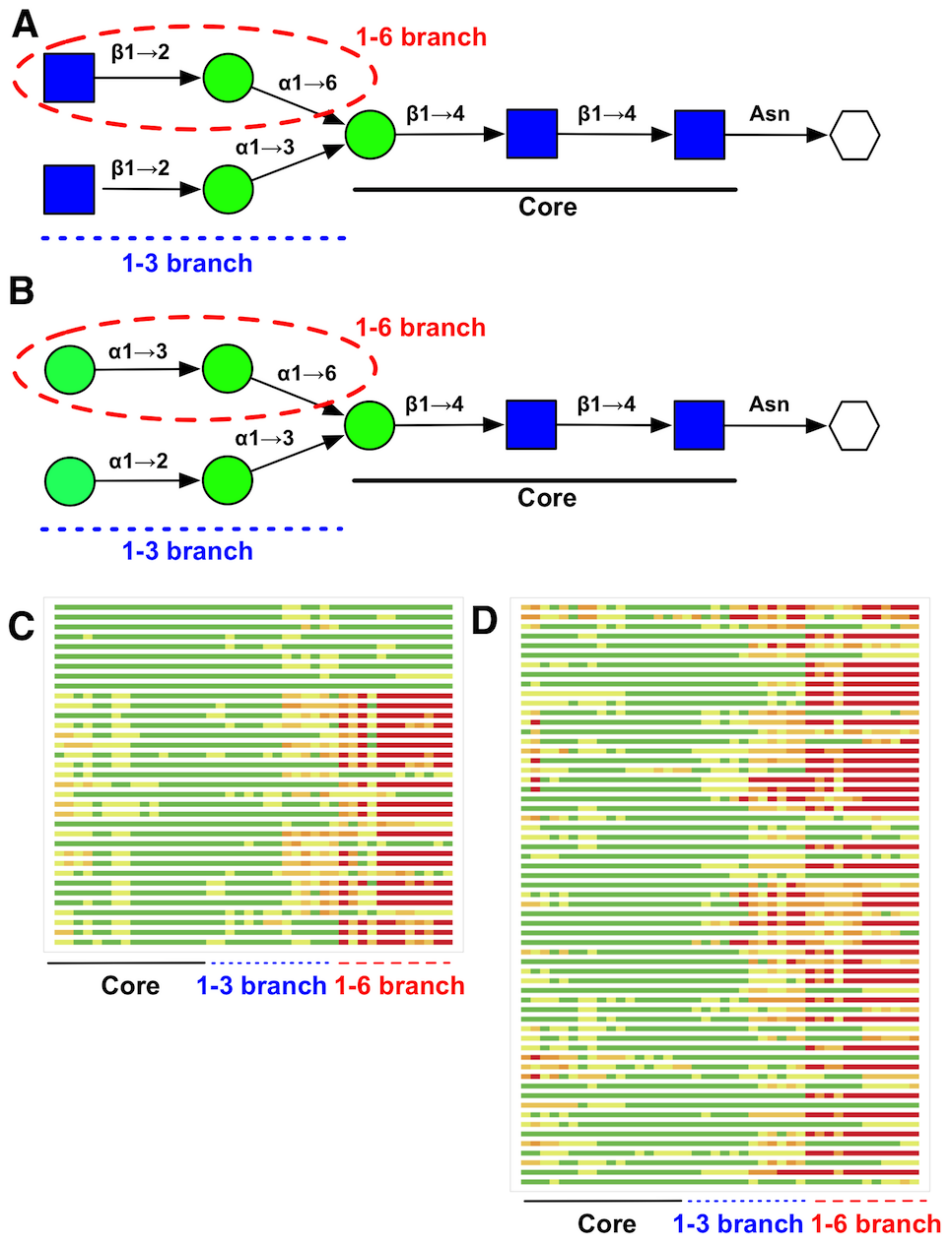
Structural flexibility within N-glycan chains. Two examples of N-glycan sequences (A and B) and their GDT chart (C for A and D for B) for the corresponding N-glycan structure pairs in the PDB. Each horizontal bars represents the distance deviation of carbohydrate ring atoms for different N-glycan structure pairs. Atoms superimposed below 1, 2, 3, and 4 Å are colored in green, light green, light orange, and orange, respectively; red for above 4 Å. The atoms in the 1–6 branch of the sequence are aligned to be at the end of the GDT charts (highlighted with dashed red lines).

To examine the flexibility of different regions of N-glycan structures, we have used the GDT chart [38]. Figure 6 shows two example N-glycan sequences and the corresponding GDT charts, where each bar represents an alignment of an N-glycan pair and the bar is colored according to how well a certain region of the sequence can be aligned each other. Clearly, the increased flexibility of terminal residues is apparent and, in particular, the residues in the 1–6 branches are even more flexible.

Non-homologous N-glycan structures in the PDB do not show a correlation with local protein structure around the glycan. There could be several factors responsible for this observation, and the accuracy of local protein structure alignment might be one important factor. To compare the similarity of local protein structure, TM-align [43] was used because the algorithm is general and performed well compared to other local structure algorithms available in our internal testing [44]. However, the TM-align algorithm was developed for comparison of global protein structure, and it is possible that the algorithm is insensitive to the structural similarities of the small number of residues around the glycan chain. Thus, further in-depth investigations with robust local structure algorithms are warranted.

### Internal substructures of N-glycan structures in the PDB are conserved

The lack of correlation between the local protein structure and non-homologous glycan structures suggests that the gapless threading approach to N-glycan modeling would be inapplicable when no homologous templates are present. It was reported that the majority of glycosylation sites are found to be in convex or flat regions of the protein surface [32]. When the N-linked oligosaccharides are situated in such regions, the terminal residues of a long oligosaccharide may not be able to interact with the protein surface residues, and experience a smaller influence of the local protein environment. Thus, local protein structure around glycan chains might have a stronger impact on the first few residues of the glycan chain rather than on the global structure.

**Figure 7:**
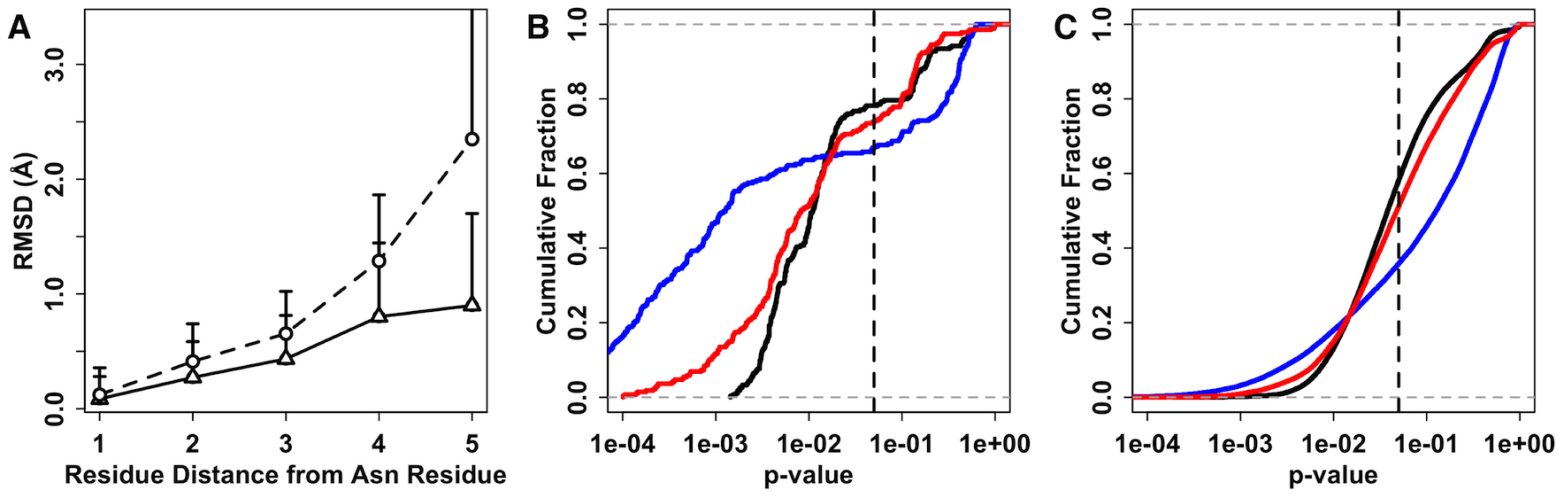
Structural similarity of N-glycan internal substructures. (A) RMSD of the internal substructures composed of residues within a certain distance from the protein. The distance is measured by the number of glycosidic linkages in a N-glycan chain including the glycosidic linkage to Asn. The lines are labeled for homologous (solid line) and non-homologous (dashed line). (B and C) Cumulative fraction of internal substructure similarity (*p*-value) for (B) homologous and (C) non-homologous glycans, respectively. The average substructure similarity of residues up to a distance of 3, 4, and 4 (without the 1–6 linkage) are colored in black, blue, and red, respectively.

Internal substructure conservation can be visualized with the two examples in Figure 6, showing that the flexibility of the carbohydrate residues increases as the residues move away from the protein. In addition, a large increase in flexibility is observed after the 1–6 linkage, which is known to be flexible. If the N-glycan substructure is more conserved, a threading or fragment assembly approach could be useful to model the N-glycan structures. To quantify the conservation of internal substructures, we compared the structural similarity of the N-glycans as a function of glycan chain length from the protein. Figure 7A shows the average RMSD of N-glycan internal substructures containing only the residues within the given residue distance from the Asn residue of the parent protein. The conservation of the internal substructure is apparent up to 3 or 4 residues away from the Asn residue. Note that N-glycan sequences can have branches, and thus, there could be more residues in a substructure within a certain residue distance. For example, in the two examples in Figure 6, there are in fact 5 sugar residues at a residue distance of 4 from Asn.

To avoid the inherent length dependence of RMSD (i.e., a smaller substructure has a smaller RMSD), RMSD values for the substructures are converted to *p*-values using the random background. Figure 7B and 7C show the cumulative fraction of the substructure similarity for homologous and non-homologous N-glycans, respectively. About 80% and 60% of the substructure up to a residue distance of 3 (black curve) show significant structural similarity for homologous and non-homologous N-glycans, respectively. The substructures are less conserved when residues up to a distance of 4 are included in the substructure (blue curve). As discussed above, due to its flexibility, the 1–6 linkage might contribute to the diversity of the N-glycan substructures more than other glycosidic linkages. Clearly, when structural similarity of substructures up to a residue distance of 4 is compared without residues linked by the 1–6 linkage (red curve), significant structural conservation is observed even for non-homologous N-glycans. This observation implies that the glycan residues closer to the protein surface have more restricted conformational space and conserved structures.

## Discussion

Elucidation of the factors influencing the conformational variability in N-glycans is essential to understand the dynamics of N-glycans and provides valuable insight into modeling and computational studies of the N-linked oligosaccharides. In this work, we have shown that the conformations of homologous N-glycans are restricted compared to the random background. About 67% of the homologous N-glycan pairs and 37% of the non-homologous N-glycan pairs show statistically significant level of structural similarity. Although excluded from the main analysis, more than 90% of highly homologous N-glycan structure pairs (protein sequence similarity ≥90%) show very significant structural similarity (Figure S5).

Why do homologous N-glycans have conserved conformations compared to the free oligosaccharides? First, protein-carbohydrate interactions may restrict the conformational freedom of the N-glycans. In addition, the shape of the local protein structure may also act as a non-specific steric barrier and restrict the N-glycans to adopt certain conformations. Lastly, crystallographic bias in the dataset could also play a role in conformational similarity of homologous N-glycan structures. Our dataset is composed of crystal structures of well-resolved N-glycan structures; hence, flexible N-glycan structures may not be included in our dataset.

Despite the biological importance of N-glycans, understanding the structure and dynamics of N-glycans is currently lacking due to the difficulties in crystallization of glycoproteins and other experimental techniques. The high level of structural similarity among the N-glycan structures found on the surface of homologous proteins strongly indicates that the comparative modeling and threading approach used in protein structure prediction [45]–[47] might perform well in glycan structure modeling if appropriate templates are present. Despite the structural similarity of N-glycans on the homologous glycoproteins, the absolute orientation of N-glycan with respect to the glycosylated Asn residue may differ because the glycosylation site are often found on the loop regions of the protein.

N-glycan modeling without good template structures appears to be challenging because of less conserved N-glycan structures found for non-homologous proteins. However, a higher level of internal substructure similarity exists even for non-homologous N-glycan pairs up to a residue distance of 4 without the 1–6 linkage. In fact, these carbohydrate structures that lie close to the protein are key determinants of the overall N-glycan orientation. Thus, a fragment assembly approach might perform well even without homologous N-glycans template structures because of this internal substructure conservation.

## Methods

### N-Glycan structure dataset

Extracting structural information of glycans from the PDB is nontrivial due to a lack of standardized nomenclature and the way the data is presented in the PDB. To recognize the PDB entries that contain carbohydrate molecules, we used *Glycan Reader* for automatic sugar identification [36]. Briefly, in *Glycan Reader*, the topologies of the molecules in the HETATM section of a PDB file are first generated using the CONECT section of the PDB file, and the candidate carbohydrate molecules (a six-membered ring for a pyranose and a five-membered ring for a furanose that are composed of carbon atoms and only one oxygen atom) are identified. For each carbohydrate-like molecule, the chemical groups attached to each position of the ring and their orientations are compared with a pre-defined table to identify the correct chemical name for the carbohydrates. Glycan chains are constructed by examining the glycosidic linkages between the carbohydrate molecules that have chemical bonds between them. As of December 2011, there were 2,517 PDB entries and 10,769 N-linked glycan chains in the RCSB database. The glycan fragment structure database, including the substructures of the original *N*-glycan chains, was generated, which resulted in a total of 48,568 N-glycan fragment chains.

From the N-glycan fragment database, we have collected glycan structures composed of more than 3 carbohydrate units. A glycan structure was excluded when its resolution was higher than 3 Å or when it had less than 20 structures in total, resulting in the 35 N-glycan fragment sequences listed in Table S1. An N-glycan structure pair is called “non-homologous” when the sequence similarity of the parent proteins is less than 30%. Because a glycoprotein can have multiple glycosylation sites in a single domain, if the distance between the backbone Cα atoms of the two glycosylated Asn residues is more than 10 Å after alignment of the glycoprotein chains using TM-align [43], the N-linked glycan structure pairs are considered “non-homologous” glycans. The rest of the N-glycan structure pairs are called “homologous” glycans. Figure 1 summarizes the protocol for building the N-glycan structure dataset.

### Generation of random glycan conformation pool

To quantify the conformational variability of the PDB N-glycan structures, it is essential to know the upper bound of the conformational variability in a given oligosaccharide. In protein structural biology, the upper bound of conformational variability is estimated by using the non-homologous protein structure pool and sequence-independent structure alignment methods [48]–[50]. However, because such sequence-independent structure alignment methods are not available for oligosaccharides, it is difficult to estimate the upper bound of the conformational variability in oligosaccharides only using the crystal structures in the PDB.

Instead of using the crystal structures directly, a conformational pool that contains diverse conformations of a specific N-glycan sequence was generated as follows. For each of the 35 N-glycan sequences, a total of 1,000,000 glycan conformations were generated in an iterative fashion. The initial structures were generated by using the *IC BUILD* command in the CHARMM biomolecular simulation program [51] according to the glycan sequence. For each iteration, a glycosidic linkage was randomly selected and a new torsion angle value was also randomly chosen based on the accessible glycosidic torsion angles of the corresponding glycosidic linkage type. If the newly generated conformation had bad contacts with neighboring atoms, the conformation was rejected and the protocol was repeated until no bad contacts were found. If a conformation had no bad contacts, the conformation was recorded and the protocol repeated until 1,000,000 conformations were generated. A bad contact was defined by the CHARMM van der Waals energy higher than 10 kcal/mol. Accessible glycosidic torsion angle values were used rather than the values observed in the PDB because the number of observations is limited for certain types of glycosidic linkages. For example, Figure S6 in Supplementary Material shows the resulting glycosidic torsion angle distributions of the N-glycan core sequence using the accessible glycosidic torsion angle values, and Figure S7 shows the torsion angle values observed in the PDB, respectively.

To construct an accessible glycosidic torsion angle map, a total of 13 adiabatic (*φ, φ, ω*) potential maps were constructed for each distinct glycosidic linkage type found in the 35 *N*-glycan sequences. For each glycosidic linkage type, a disaccharide connected by the corresponding glycosidic linkage type was generated by CHARMM [51], and the CHARMM carbohydrate force field [52]–[54] was used to evaluate the energy. The adiabatic map was generated by evaluating the energy over a grid of glycosidic torsion angles with a grid spacing of 5°, resulting in a total of 373,248 grid points for (1→6) linkages (*φ, φ, ω*) and 5,184 grid points for the rest of the glycosidic linkages (*φ, φ*). At each grid point, the conformations were minimized with the dielectric-screened Coulombic electrostatic and Lennard-Jones potential energy while the glycosidic torsion angles were restrained and a harmonic restraint potential was applied to the carbohydrate rings to prevent the distortion of the ring geometry. The generated adiabatic potential energy map was converted to a torsion angle probability map using the Boltzmann distribution. Finally, the resulting distribution was compared with the glycosidic torsion angles observed in the PDB using the *Glycan Fragment DB* [55], available at www.glycanstructure.org. The glycosidic torsion angle probability maps and the observations in the PDB matched well in general. However, the torsion angle probability map was clearly more restricted (data not shown). To remedy the restricted conformational space, glycosidic torsion angle pairs having probability above 0.0001 were considered “accessible”; this covers on average about 65% of the observed glycosidic torsion angles in the PDB.

### Structural similarity of N-glycan and its statistical significance

The N-glycan structural similarity was measured by calculating pairwise RMSD in the following three different ways: First, the heavy atoms in the carbohydrate ring (C_1_, C_2_, C_3_, C_4_, C_5_, and O_5_) were used for the alignment of two N-glycan structures and in the RMSD calculation. Second, to examine the variability of N-glycan orientations with respect to the protein, the heavy atoms of glycosylated Asn residues were used to define the alignment, and then the Euclidean distance of the N-glycan structures was calculated using the carbohydrate ring heavy atoms. Third, many crystal structures only have a few residues at the glycosylation site due to difficulties associated with glycan crystal structure determination, and these partial glycan structures can be used to model the rest of a full glycan structure. To examine the efficacy of such an approach in obtaining a better N-glycan orientation with respect to the protein, the carbohydrate ring heavy atoms of the first two residues were used for the alignment of N-glycan structures, and then the Euclidean distance of the N-glycan structures was calculated using the ring heavy atoms excluding the first two residues.

The statistical significance of the structural similarity between two glycan structures was estimated by comparing the structural similarity of 124,750 random glycan structure pairs for each N-glycan sequence. The structural similarity of random glycan structure pairs was calculated by the identical procedure described above. Using the statistical model, *p*-values of the corresponding structural similarity measure can be calculated. This allows us to compare structural similarity across different glycan sequences and lengths. Each RMSD distribution for each glycan sequence was modeled by the generalized extreme value distribution,
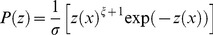
(1)where 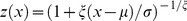. The variable 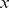 represents the RMSD of a structure pair; 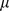, 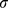, and 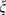 are the location, scale, and shape parameters, respectively. These parameters were obtained through the maximum likelihood estimates by the EVD package in R (http://www.r-project.org). 35 sets of determined parameters are given in Table S2 and the fitting results are shown in Figure S2. The resulting correlational coefficients (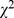) are generally good except for a few sequences. The correlation coefficients improved when more “liberal” protocols were used (e.g., when the glycosidic linkage was not restricted and larger energy cutoff values were used to define bad contacts; data not shown). However, such protocols may produce unrealistic random glycan conformers and are not used in this work. The *p*-value of a glycan structure pair from the PDB having RMSD values smaller than the random glycan conformation background was calculated by
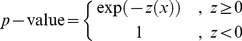
(2)


### Local structure alignment and statistical significance

The local protein structures are defined for protein residues having any heavy atoms within 6 Å from any glycan heavy atoms. The local protein structures were derived from the PDB structure files in our dataset, and the TM-align algorithm [43] was used to compare the structural similarity of a given local protein structure pair. Any local protein structures having less than 5 residues were excluded. The TM-scores calculated by TM-align were normalized by the length of the smaller structure. To estimate statistical significance, we have derived a random local protein structure pool using the N-linked glycoproteins in the PDB. Briefly, from the PDB, a non-redundant N-linked glycoprotein structure list having at least one carbohydrate residue and protein sequence similarity less than or equal to 30% were generated. A random local protein structure pool was derived from the protein residues having any heavy atoms within 6 Å from any of the carbohydrate heavy atoms. The TM-align algorithm was used to calculate the distribution of TM-scores from the random local protein structure pairs. The calculated TM-score distribution was fit using the generalized extreme distribution (Eq. (1)), and the *p*-values of having TM-scores larger than the random background were estimated using Eq. (2).

Although there are several local structure alignment tools available [56]–[58], it was difficult to directly utilize them in this study because many of them are highly customized to specific domains, such as a protein-protein interface or protein-ligand interface. Thus, we used TM-align [43] to compare local structure similarity. Although TM-align is not designed to compare local structure similarity, it performed well in our internal testing and correctly found most homologous glycoproteins having similar local protein Cα structures; also see ref for protein local structure comparisons [44].

### Structural similarity of internal substructure and the statistical significance

The residue distance is defined as the minimum number of glycosidic linkages between carbohydrate monomers, including the glycosidic linkage to Asn. For each of 35 N-glycan sequences, three types of internal substructures were generated; a) residue distance up to 3, b) residue distance up to 4, and c) residue distance up to 4, excluding residues linked by the 1–6 linkage. Then, the RMSD of substructures were measured after alignment using the carbohydrate ring atoms in the substructure. To estimate the statistical significance of the internal substructures, the random glycan internal structure pool was generated for each of three different types of substructures. The resulting random background distributions were fit using Eq. (1) and p-values were calculated using Eq. (2).

## Supporting Information

Figure S1 Overlay of the N-glycan core structures from the various IgG1 structures from the PDB. The PDB entries used in this overlay are 3AVE, 3AY4, 3C2S, 3D6G, 3DO3, 2DTS, 3FJT, 1H3X, 1I1A, 1I1C, 1L6X, 1OQO, 2QL1, 2RGS, 3SGJ, 3SGK, and 2VUO.
(TIFF)

Figure S2 The comparison of the original RMSD distributions (dashed line) and the fitted generalized extreme distributions (solid line). The numbers on each plots represents the sequence identification number used in Table S1.
(TIFF)

Figure S3 Correlation between the sequence similarity and the structural similarity (*p*-value). The box represents the range between the first and third quartiles of the distribution and the thick horizontal lines represent the median of the distribution. The open circles are outliers.
(TIFF)

Figure S4 Structural similarity of N-glycans using the GDT-TS score. The GDT-TS score distributions are for the homologous (red) and non-homologous (blue) structure pairs. The GDT-TS score is defined as GDT-TS = (P0.5+P1+P2)/3 where P*X* is the fraction of atoms that can be superimposed with corresponding cutoffs of *X* = 0.5, 1, and 2 Å.
(TIFF)

Figure S5 Cumulative fraction of structure similarity of N-glycan pairs whose parent proteins have sequence similarity greater than or equal to 90%.
(TIFF)

Figure S6 Glycosidic torsion angle distributions from the random glycan conformation pool for the N-glycan core sequence. 1,000,000 conformations were generated by assigning randomly chosen torsion angle values from the accessible torsion angles of the corresponding glycosidic linkage type. The following glycosidic torsion angle definitions are used; O_5_-C_1_-O_1_-C′_x_ (*φ*), C_1_-O_1_-C′_x_-C′_x-1_ (ψ), and O_1_-C′_6_-C′_5_-O′_5_ (*ω*).
(TIFF)

Figure S7 Glycosidic torsion angle distributions for the corresponding glycosidic linkage type (disaccharide) observed in the PDB. The *Glycan Fragment DB* (www.glycanstructure.org/fragment-db) was used to collect the glycosidic torsion angle distribution in the PDB. The following glycosidic torsion angle definitions are used; O_5_-C_1_-O_1_-C′_x_ (*φ*), C_1_-O_1_-C′_x_-C′_x-1_ (ψ), and O_1_-C′_6_-C′_5_-O′_5_ (*ω*).
(TIFF)

Table S1 List of N-linked oligosaccharide sequences used in this study. The nomenclature for glycan representation is adopted from ref. [1]: blue square for *N-acetyl glucose*, green circle for *mannose*, red triangle for *fucose*, yellow star for xylose. The number of (non-redundant) homologous and non-homologous N-glycan structure pairs are given for each N-glycan sequence.
(DOCX)

Table S2 Parameters for the generalized extreme value distributions.
(DOCX)
