# Supplementary figures and images for: Restricted N-glycan Conformational Space in the PDB and Its Implication in Glycan Structure Modeling
Source: PLoS Comput Biol. 2013 Mar 14;9(3):e1002946. doi: 10.1371/journal.pcbi.1002946 (PMC3597548; doi:10.1371/journal.pcbi.1002946)

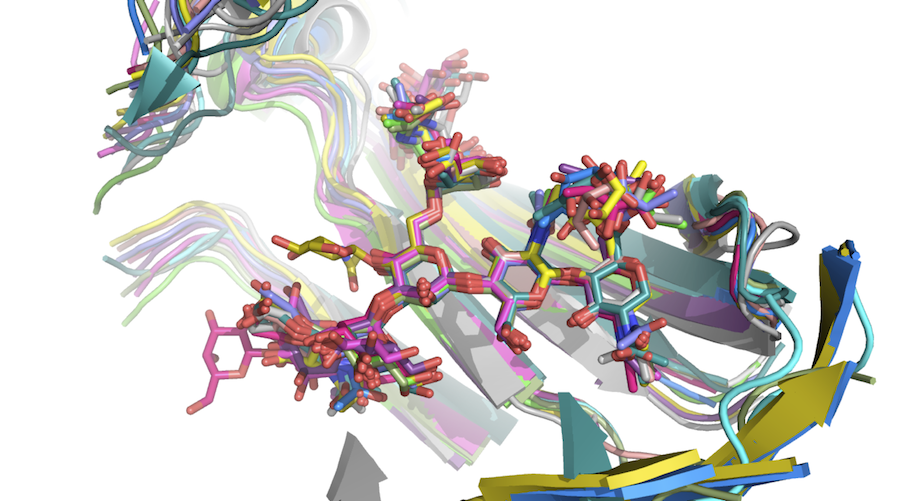

Supplement: Figure S1 — Overlay of the N-glycan core structures from the various IgG1 structures from the PDB. The PDB entries used in this overlay are 3AVE, 3AY4, 3C2S, 3D6G, 3DO3, 2DTS, 3FJT, 1H3X, 1I1A, 1I1C, 1L6X, 1OQO, 2QL1, 2RGS, 3SGJ, 3SGK, and 2VUO. (TIFF) [file pcbi.1002946.s001.tif]

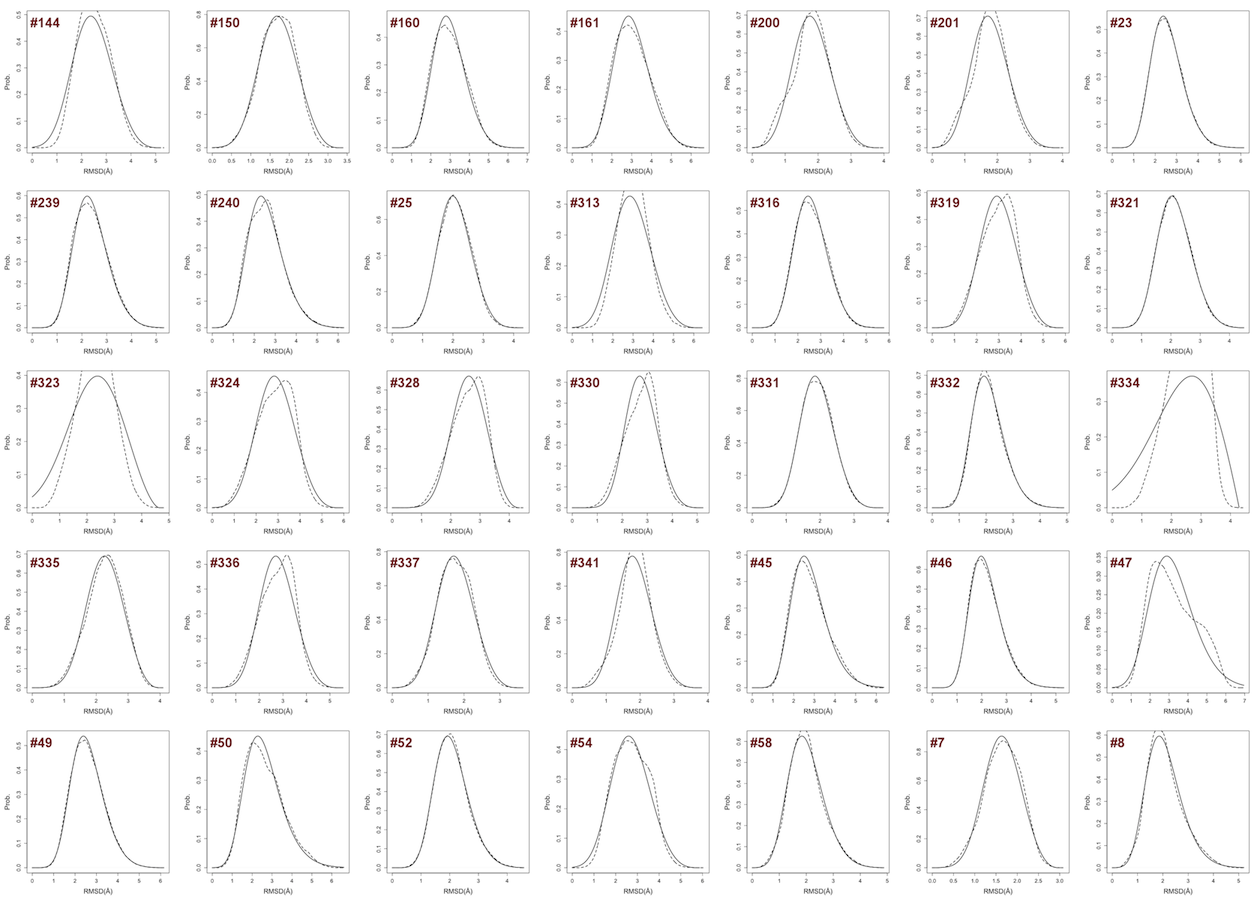

Supplement: Figure S2 — The comparison of the original RMSD distributions (dashed line) and the fitted generalized extreme distributions (solid line). The numbers on each plots represents the sequence identification number used in Table S1. (TIFF) [file pcbi.1002946.s002.tif]

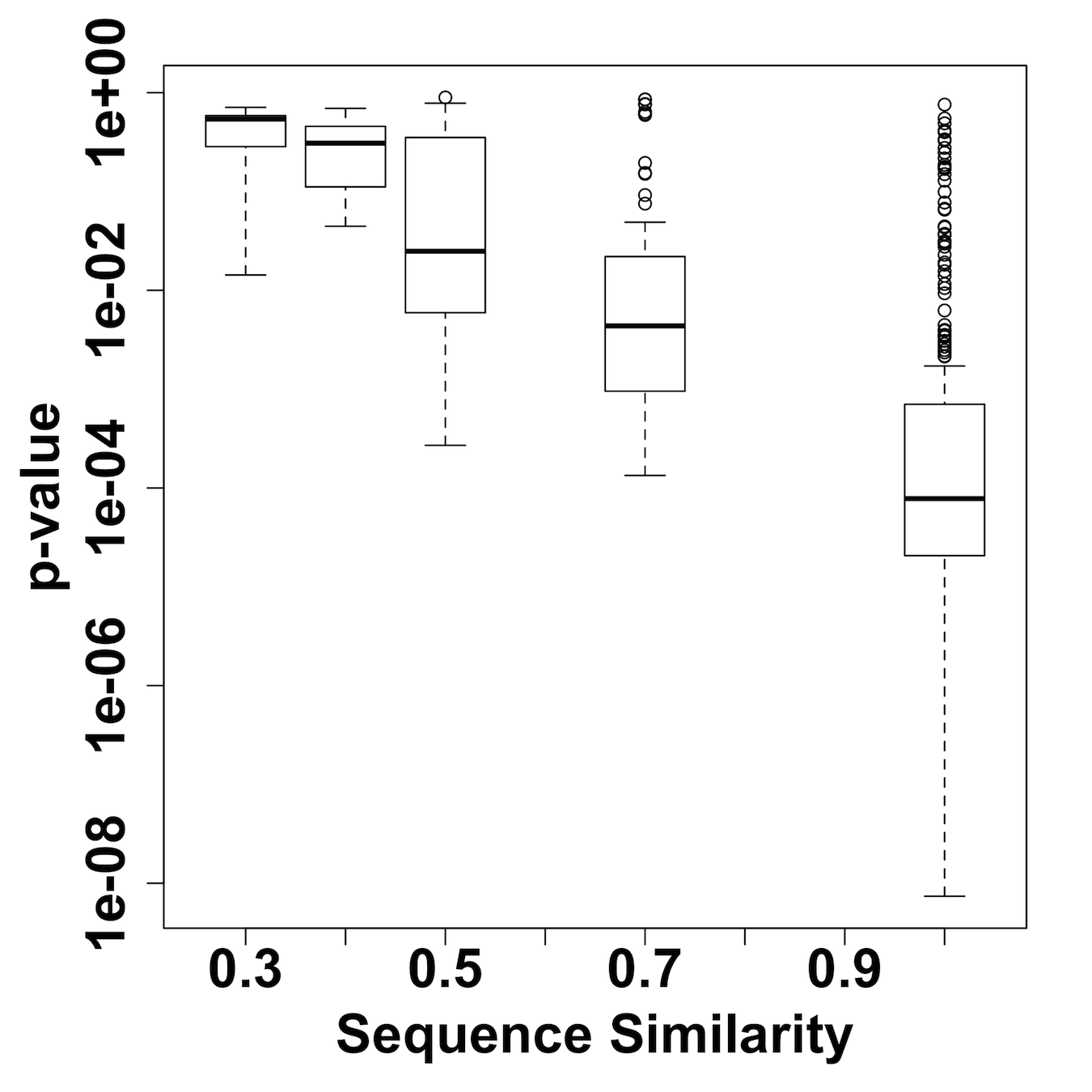

Supplement: Figure S3 — Correlation between the sequence similarity and the structural similarity (p-value). The box represents the range between the first and third quartiles of the distribution and the thick horizontal lines represent the median of the distribution. The open circles are outliers. (TIFF) [file pcbi.1002946.s003.tif]

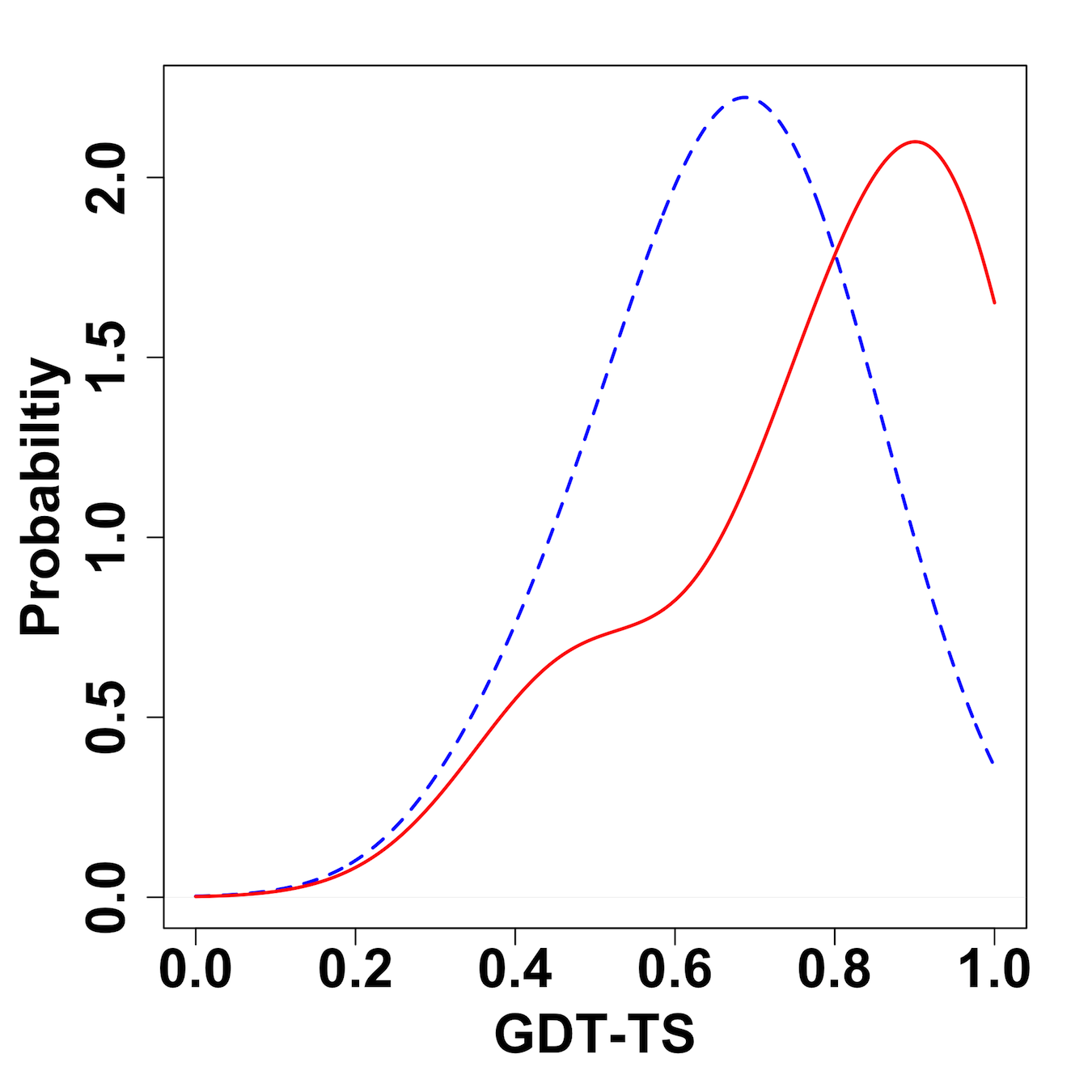

Supplement: Figure S4 — Structural similarity of N-glycans using the GDT-TS score. The GDT-TS score distributions are for the homologous (red) and non-homologous (blue) structure pairs. The GDT-TS score is defined as GDT-TS = (P0.5+P1+P2)/3 where PX is the fraction of atoms that can be superimposed with corresponding cutoffs of X = 0.5, 1, and 2 Å. (TIFF) [file pcbi.1002946.s004.tif]

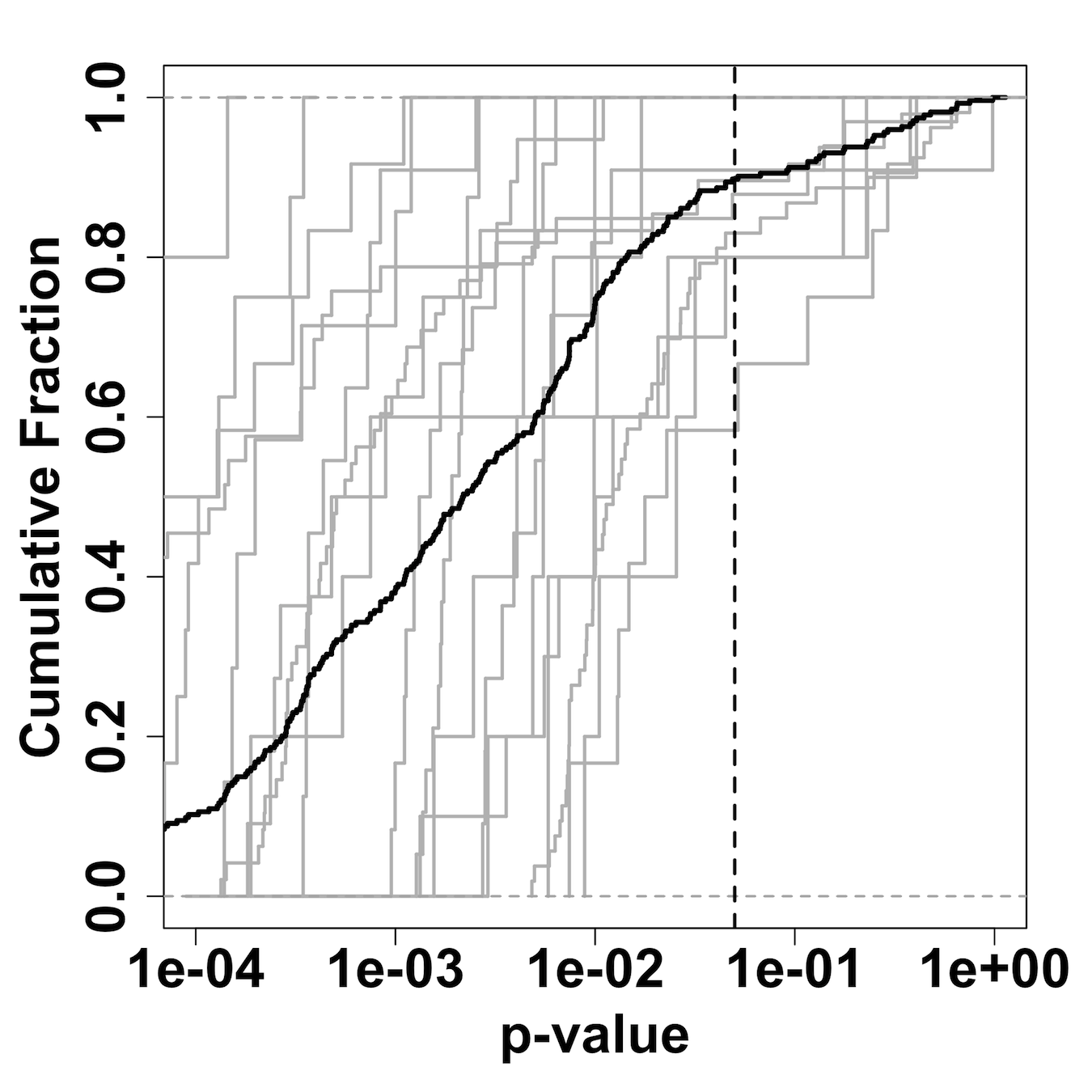

Supplement: Figure S5 — Cumulative fraction of structure similarity of N-glycan pairs whose parent proteins have sequence similarity greater than or equal to 90%. (TIFF) [file pcbi.1002946.s005.tif]

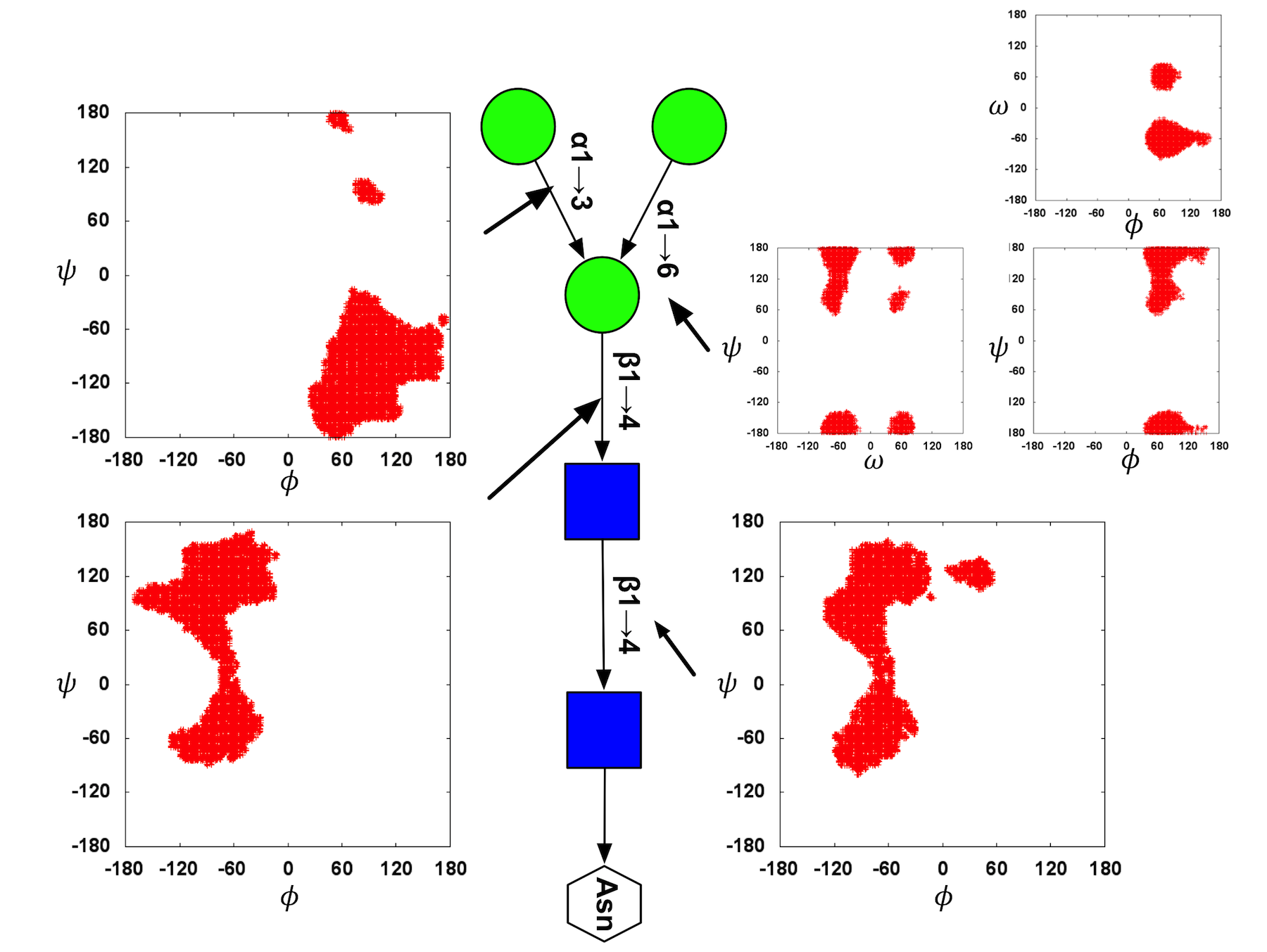

Supplement: Figure S6 — Glycosidic torsion angle distributions from the random glycan conformation pool for the N-glycan core sequence. 1,000,000 conformations were generated by assigning randomly chosen torsion angle values from the accessible torsion angles of the corresponding glycosidic linkage type. The following glycosidic torsion angle definitions are used; O5-C1-O1-C′x (φ), C1-O1-C′x-C′x-1 (ψ), and O1-C′6-C′5-O′5 (ω). (TIFF) [file pcbi.1002946.s006.tif]

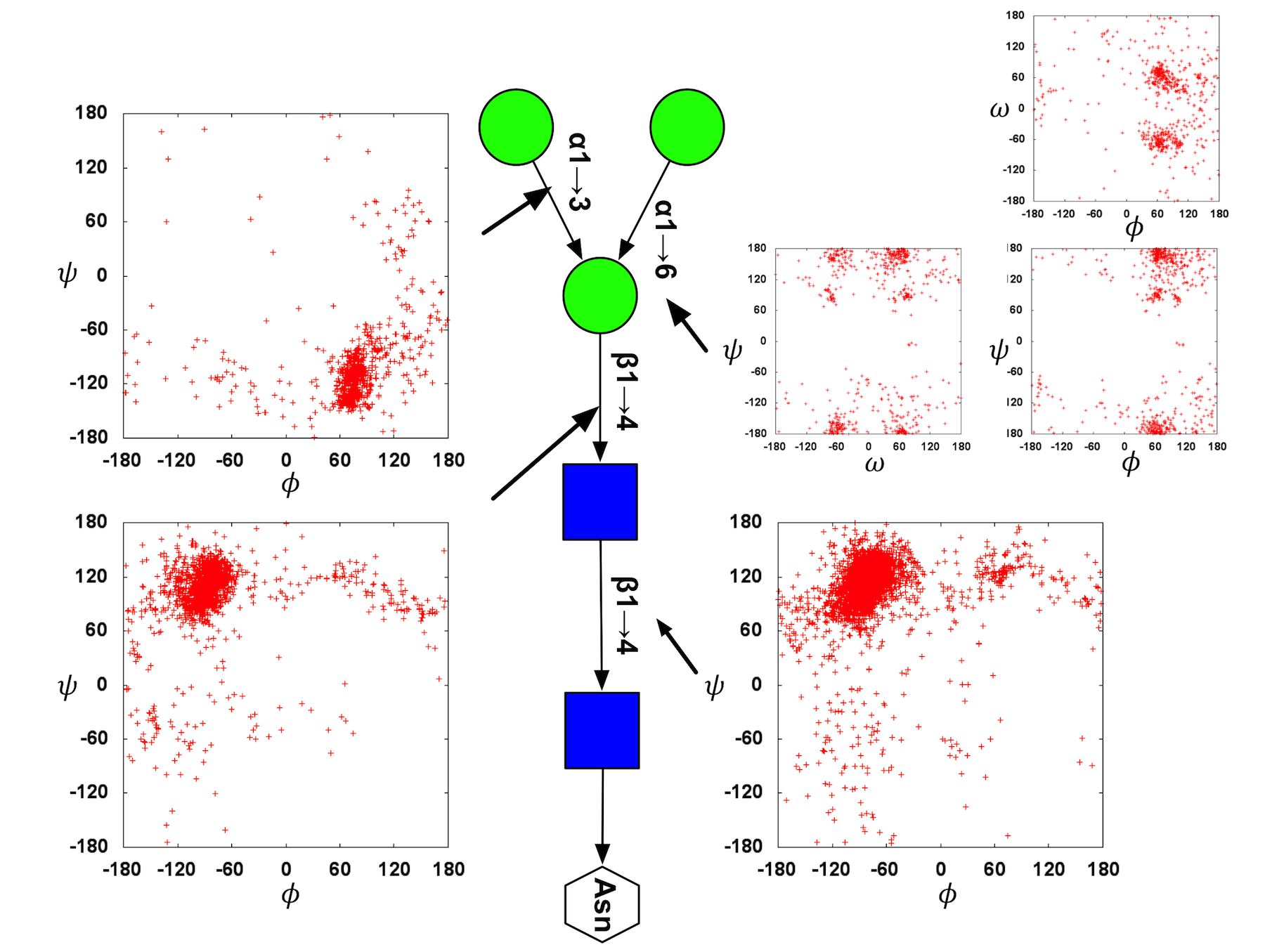

Supplement: Figure S7 — Glycosidic torsion angle distributions for the corresponding glycosidic linkage type (disaccharide) observed in the PDB. The Glycan Fragment DB (www.glycanstructure.org/fragment-db) was used to collect the glycosidic torsion angle distribution in the PDB. The following glycosidic torsion angle definitions are used; O5-C1-O1-C′x (φ), C1-O1-C′x-C′x-1 (ψ), and O1-C′6-C′5-O′5 (ω). (TIFF) [file pcbi.1002946.s007.tif]

| # | Sequence | Length | # of  homologous N-glycan pairs | # of  non-homologous N-glycan pairs |
| --- | --- | --- | --- | --- |
| 45 | 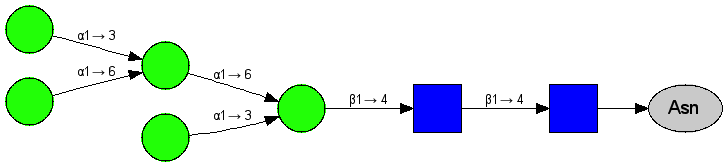 | 7 | 7 | 414 |
| 313 | 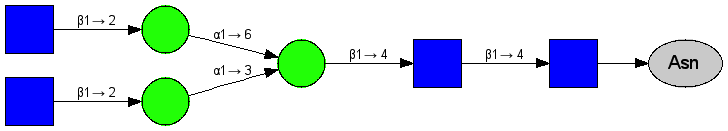 | 7 | 9 | 26 |
| 161 | 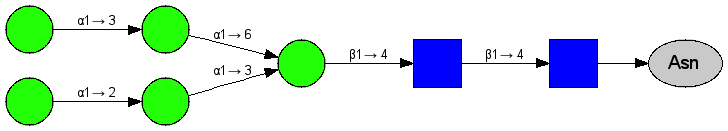 | 7 | 2 | 59 |
| 160 | 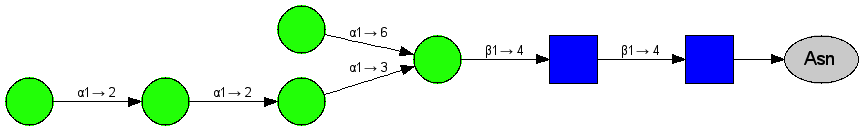 | 7 | 0 | 29 |
| 49 | 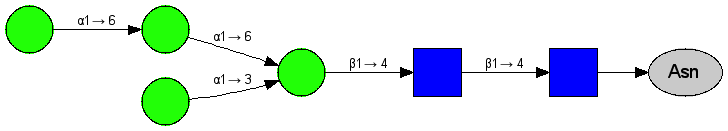 | 6 | 11 | 502 |
| 47 | 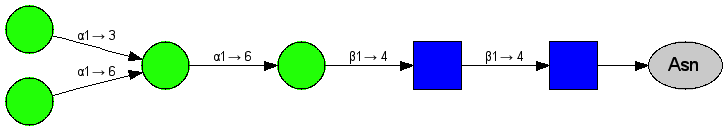 | 6 | 7 | 651 |
| 46 | 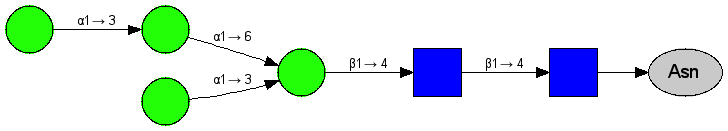 | 6 | 8 | 680 |
| 330 | 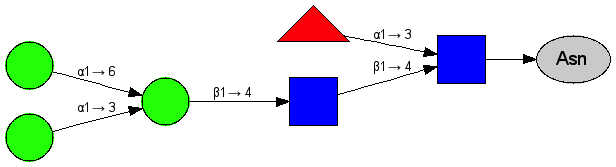 | 6 | 0 | 19 |
| 328 | 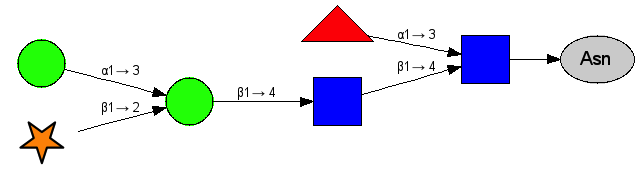 | 6 | 0 | 3 |
| 319 | 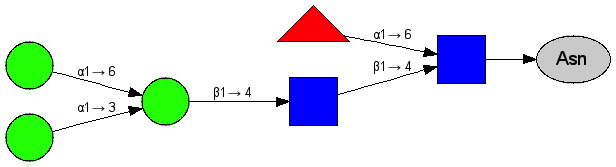 | 6 | 1 | 40 |
| 316 | 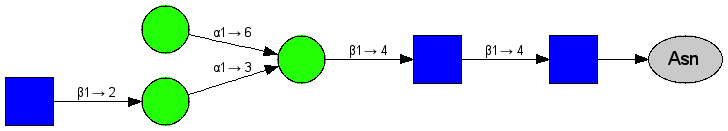 | 6 | 9 | 35 |
| 239 | 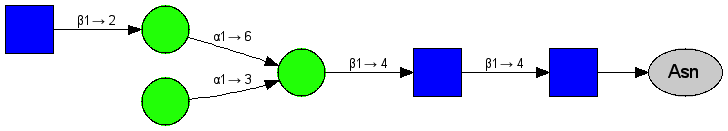 | 6 | 14 | 63 |
| 23 | 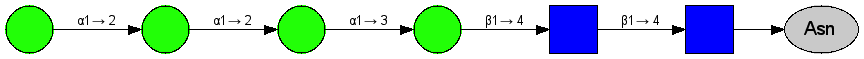 | 6 | 0 | 38 |
| 144 | 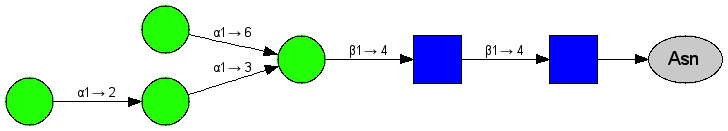 | 6 | 11 | 168 |
| 54 | 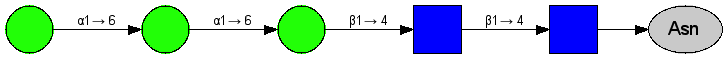 | 5 | 12 | 95 |
| 52 | 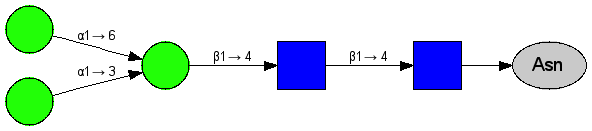 | 5 | 42 | 421 |
| 50 | 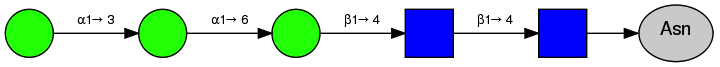 | 5 | 9 | 44 |
| 336 | 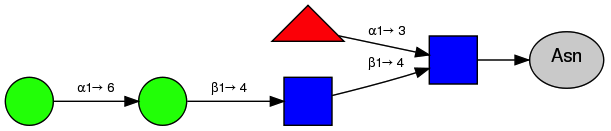 | 5 | 0 | 83 |
| 335 | 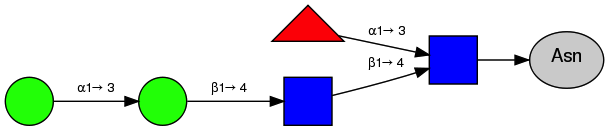 | 5 | 0 | 116 |
| 334 | 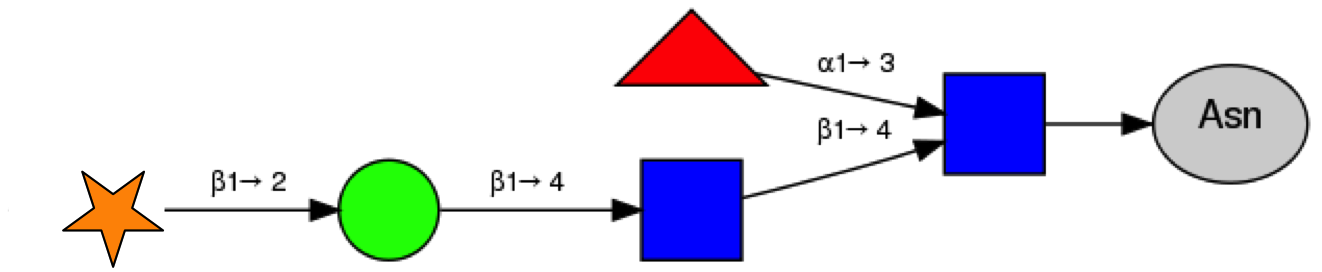 | 5 | 0 | 24 |
| 332 | 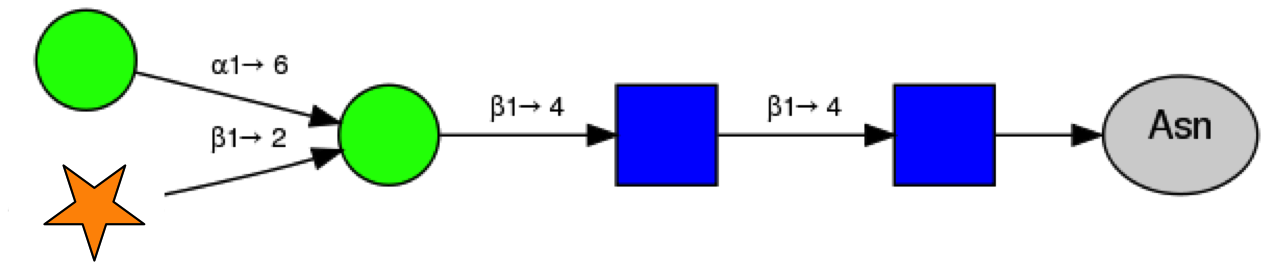 | 5 | 0 | 23 |
| 331 | 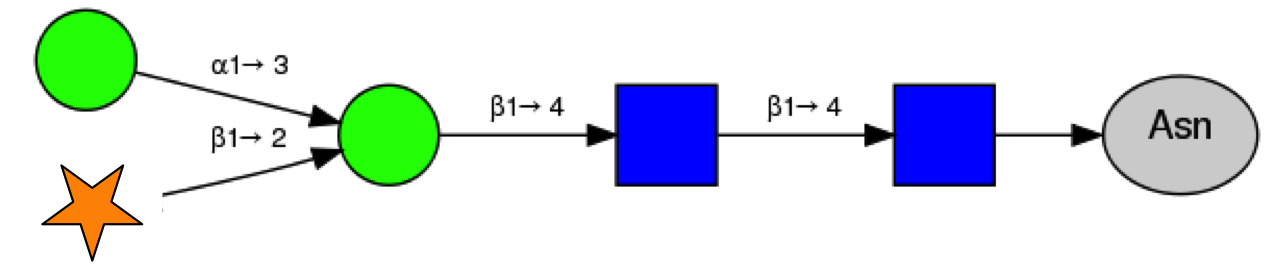 | 5 | 0 | 29 |
| 324 | 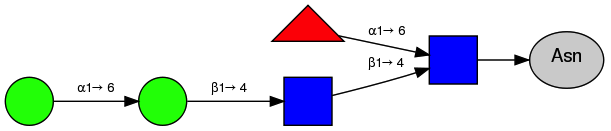 | 5 | 1 | 50 |
| 323 | 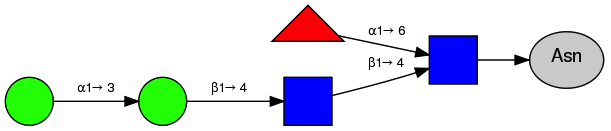 | 5 | 2 | 47 |
| 321 | 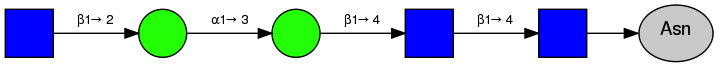 | 5 | 9 | 1230 |
| 25 | 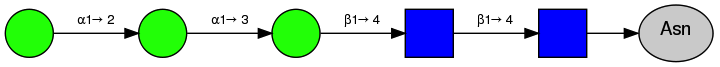 | 5 | 14 | 5128 |
| 240 | 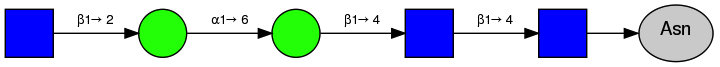 | 5 | 14 | 760 |
| 8 | 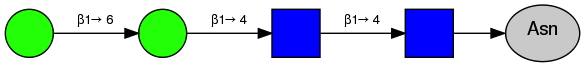 | 4 | 0 | 156 |
| 7 | 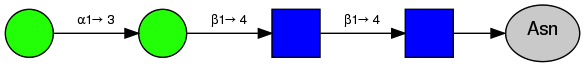 | 4 | 47 | 11578 |
| 58 | 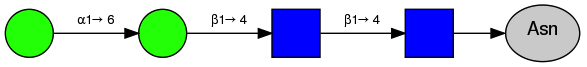 | 4 | 52 | 9544 |
| 341 | 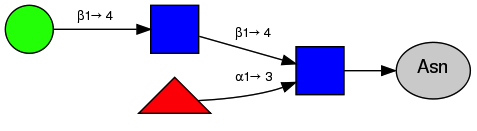 | 4 | 2 | 389 |
| 337 | 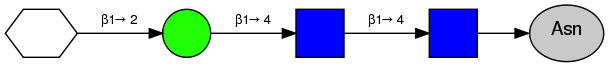 | 4 | 1 | 93 |
| 201 | 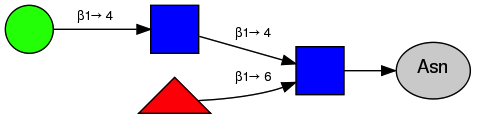 | 4 | 2 | 52 |
| 200 | 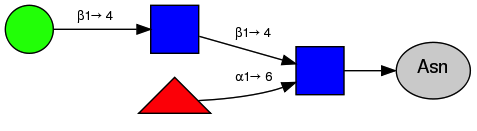 | 4 | 3 | 496 |
| 150 | 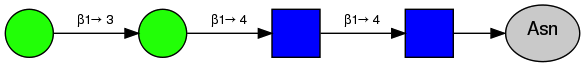 | 4 | 0 | 248 |

Supplement: Table S1 — List of N-linked oligosaccharide sequences used in this study. The nomenclature for glycan representation is adopted from ref. [1]: blue square for N-acetyl glucose, green circle for mannose, red triangle for fucose, yellow star for xylose. The number of (non-redundant) homologous and non-homologous N-glycan structure pairs are given for each N-glycan sequence. (DOCX) [file pcbi.1002946.s008.docx]
