# Supplementary material for: Restricted N-glycan Conformational Space in the PDB and Its Implication in Glycan Structure Modeling
Source: PLoS Comput Biol. 2013 Mar 14;9(3):e1002946. doi: 10.1371/journal.pcbi.1002946 (PMC3597548; doi:10.1371/journal.pcbi.1002946)
Supplement: Table S2 — Parameters for the generalized extreme value distributions. (DOCX) [file pcbi.1002946.s009.docx]

| # | $\mu$ | $\sigma$ | $\xi$ | $\chi^{2}$ |
| --- | --- | --- | --- | --- |
| 45 | 2.42 | 0.74 | -0.10 | 1.4 |
| 313 | 2.60 | 0.89 | -0.24 | 21.6 |
| 161 | 2.67 | 0.83 | -0.18 | 1.1 |
| 160 | 2.63 | 0.78 | -0.18 | 0.9 |
| 49 | 2.28 | 0.69 | -0.14 | 0.1 |
| 47 | 2.73 | 1.04 | -0.13 | 14.5 |
| 46 | 1.91 | 0.55 | -0.10 | 0.19 |
| 330 | 2.54 | 0.60 | -0.23 | 4.60 |
| 328 | 2.42 | 0.57 | -0.30 | 6.70 |
| 319 | 2.68 | 0.78 | -0.26 | 2.87 |
| 316 | 2.31 | 0.67 | -0.18 | 0.30 |
| 239 | 2.11 | 0.62 | -0.15 | 0.40 |
| 23 | 2.27 | 0.66 | -0.14 | 0.08 |
| 144 | 2.14 | 0.77 | -0.25 | 27.58 |
| 54 | 2.36 | 0.85 | -0.24 | 7.16 |
| 52 | 1.84 | 0.54 | 0.15 | 0.25 |
| 50 | 2.21 | 0.82 | -0.09 | 2.02 |
| 336 | 2.50 | 0.71 | -0.24 | 3.76 |
| 335 | 2.07 | 0.56 | -0.29 | 1.12 |
| 334 | 2.00 | 1.16 | -0.51 | 507.15 |
| 332 | 1.84 | 0.53 | -0.13 | 0.35 |
| 331 | 1.73 | 0.46 | -0.23 | 0.17 |
| 324 | 2.59 | 0.83 | -0.26 | 3.97 |
| 323 | 1.96 | 1.00 | -0.37 | 263.86 |
| 321 | 1.92 | 0.55 | -0.19 | 0.10 |
| 25 | 1.86 | 0.51 | -0.22 | 0.45 |
| 240 | 2.21 | 0.75 | -0.13 | 0.48 |
| 8 | 1.78 | 0.62 | -0.11 | 1.01 |
| 7 | 1.50 | 0.42 | -0.28 | 1.96 |
| 58 | 1.74 | 0.59 | -0.14 | 0.57 |
| 341 | 1.65 | 0.49 | -0.22 | 5.00 |
| 337 | 1.58 | 0.49 | -0.25 | 1.51 |
| 201 | 1.56 | 0.53 | -0.22 | 3.76 |
| 200 | 1.60 | 0.55 | -0.24 | 4.02 |
| 150 | 1.53 | 0.48 | -0.27 | 4.13 |
